# Schizophrenia and medication adherence among the population in Morocco: a cross-sectional study at the University Psychiatric Center of Casablanca

**DOI:** 10.11604/pamj.2024.48.123.39645

**Published:** 2024-07-22

**Authors:** Imane Salihi, Nadia Attouche, Gladys Tsoumbou Bakana, Samira Nani, Mohamed Agoub, Khadija Mchichi Alami

**Affiliations:** 1 Laboratory of Clinical Neurosciences and Mental Health, Hassan II University, Casablanca, Morocco,; 2 Laboratory of Community Medicine, Hassan II University, Casablanca, Morocco

**Keywords:** Adherence, non-adherence, schizophrenia

## Abstract

**Introduction:**

schizophrenia is a chronic, disabling, and serious disease. It represents a challenge because of its prevalence and its consequences in terms of morbidity and mortality for patients, but also for their families and society. Patients often fail to adhere to their treatment, and this has a severe negative effect on the prognosis of the disease. Thus, the identification of the predictive factors influencing this compliance is very important for adequate management and a favorable evolution. The aim of the study is to assess the predictive factors of non-adherence in patients with schizophrenia.

**Methods:**

a cross-sectional study of 320 patients diagnosed with schizophrenia according to the DSM-5 criteria, was conducted at the University Psychiatric Centre of Casablanca, Morocco. Epidemiological, clinical, and therapeutic data were collected using a hetero-questionnaire, while medication adherence was assessed using the Medication Adherence Rating Scale (MARS). The positive and negative symptoms scale (PANSS) was used to assess the severity of symptoms in patients with schizophrenia.

**Results:**

in our study, the total sample comprised 320 (100%) patients, classified into two groups: 82 (25.62%) were categorized as adherent, while 238 (74.38%) were non-adherent, and 72% were male. The non-adherent group was young (p=0.003), and a significant proportion had no educational background (p=0.015), lived alone (p=0,001), in urban areas (p=0.031), non-regular follow-up (p=0.045) and had a toxic history (p=0.0001), early age of onset of the disease (p=0.002). Moreover, this group exhibited more severe schizophrenic symptoms (p=0.02), lacked insight into their condition (p=0.046), and predominantly used typical antipsychotics (p=0.019) with a high frequency of intake (p=0.0001). Sedation emerged as a predominant side effect (p=0.036) of treatment. Notably, a high frequency of hospitalizations (p=0.005) exhibited a strong association with medication non-adherence. The mean age of the sample was 32.9 years (standard deviation: 10.8), with a mean age of disease onset reported at 25.5 years (standard deviation=4.9).

**Conclusion:**

this study highlights the prevalence of non-adherence among patients with schizophrenia, with significant associations observed with demographic factors, the severity of symptoms, treatment patterns, and hospitalization frequency, emphasizing the urgent need for tailored interventions to enhance medication adherence and improve patient outcomes in managing schizophrenia.

## Introduction

Schizophrenia is a mental disorder characterized by disruptions in thought processes, perceptions, emotional responsiveness, and social interactions, it affects 0.33% to 0.75% of the world´s population [1]. It is typically diagnosed in the late teens years to early thirties and tends to emerge earlier in males (late adolescence - early twenties). More subtle changes in cognition and social relationships may precede the diagnosis, often by years. Childhood-onset cognitive impairment and unusual behaviors in schizophrenia underscore the importance of early interventions for improved long-term outcomes [2,3]. The data on the prevalence of lack of adherence among subjects with schizophrenia vary markedly in the different studies. This fact corresponds mainly to the absence of clearly established defining criteria and the difficulties in its evaluation. However, it is estimated that the rates of lack of adherence range between 20% and 89%, so there is no doubt that the prevalence of this phenomenon is very high among subjects with schizophrenia [4].

Non-adherence with antipsychotic medication leads to relapse for patients in remission and persistent symptoms for those with existing symptoms and in both cases can cause multiple patient and service costs. The costs of non-adherence were demonstrated in a 3-year, prospective, observational study of schizophrenia in the USA in which a composite measure of patient-reported adherence and medication possession ratio (MPR) was used to determine adherence [5]. Non-adherence was associated with a significantly higher rate of psychiatric hospitalization, use of emergency psychiatric services, arrest, violence, victimization, substance use, and more alcohol-related problems and plus poorer mental functioning and poorer life satisfaction. Non-adherence in the first year predicted significantly poorer outcomes in the following 2 years. A 3-year, prospective, observational, European study of outpatients with schizophrenia found that non-adherence was significantly associated with an increased risk of relapse, hospitalization, and suicide attempts [6]. An association between antipsychotic non-adherence and an increased rate of self-harm [7] and suicide [8] has been reported in other studies. A recent analysis from the Cost Utility of the Latest Antipsychotic Drugs in Schizophrenia Study (CUTLASS) clinical trial in the UK showed that improved antipsychotic adherence led to improved quality of life [9].

This study aimed to determine the prevalence of medication non-adherence among individuals with schizophrenia and to evaluate the associated factors.

## Methods

**Study design and setting:** this study is a cross-sectional with an analytical focus and prospective recruitment, aimed at achieving the above-mentioned objectives. The work was carried out over a period of 19 months, from the bibliographical research (September 2018 - April 2019), done at the Epidemiology Department to the data collection (May 2019 - January 2020), and the finalization (February 2020 - May 2020), at the University Psychiatric Center of Casablanca, Morocco.

**Study population:** for the calculation of the sample the prevalence of schizophrenia in the literature (1%) and the desired precision (5%) had to be taken into account (Sample size = 320).

**Inclusion criteria:** age > 18-years-old, informed patients, and volunteers, patients with a diagnosis of schizophrenia according to DSM-5 criteria, any clinical form, and patients already on neuroleptic treatment for at least a year.

**Exclusion criteria:** refusal of consent for the study, uncooperative patients, schizophrenia with neurological comorbidity or delayed mental, a schizoaffective, or depressive, or a bipolar disorder with psychotic manifestations.

**Data collection method:** for each patient, a form has been drawn up containing the socio-demographic characteristics of the patient, personal and family history, the regularity of follow-up assessed on the criterion of no delay of more than one week in appointments during the three months prior to the interview, characteristics of the disease, and symptoms and their characteristics by the evaluation of the positive and negative symptoms scale (PANSS). As for the degree of a patient´s awareness of the disease or the Insight, the Birchwood Scale was used to help with the study. To assess each patient's adherence, the (MARS) Medication Adherence Rating Scale was used. This is a self-administered questionnaire with 10 items to which patients answer yes or no and the result is expressed as a total score between 0 and 10. Finally, plausible causes of treatment discontinuation, which are widely answered in the literature, were sought by means of a hetero questionnaire.

**Statistical analysis:** data were collected and coded in Microsoft Excel. Statistical analysis was performed using SPSS statistical analysis software: Statistical Package for Social Sciences (SPSS) for Windows, version 13.0 (SPSS Inc., Chicago). The descriptive analysis consisted of calculating the absolute and relative frequencies for the qualitative variables, and the positioning and dispersion parameters for the quantitative variables (mean, standard deviation). The Chi-square test was used for qualitative data. Student´s t-test was used for quantitative data. P value < 0.05 was considered statistically significant.

**Ethical considerations:** patients diagnosed with schizophrenia according to the DSM-5 criteria, followed up on an outpatient basis, we approached to participate in the research by revealing the research protocol to them. Consent was taken to include the patients in the study. The subjects were assured that their identity would remain anonymous and their responses would be kept confidential. The right was given to participants to decide whether they wished to be part of the research or not.

## Results

The study of 320 recruited patients revealed a high prevalence of medication non-adherence at 74.38%. Factors associated with non-adherence included being young (p=0.03, OR=0.9615, 95% CI=-7.3483 - -1.5511), living alone (p=0.001, OR=0.1443, 95% CI=0.0728 - 0.2879), and having no educational level (p=0.015, OR=1.0392, 95% CI=0.1862 - 0.5067). The mean age of the participants was 32.9 years. Urban living was also associated with non-adherence (p=0.031, OR=0.0635, 95% CI=0.2505 - 0.6656). Substance use history, including tobacco, alcohol, and cannabis, showed significant associations with non-adherence (p=0.0001, OR=3.259, 95% CI=1.929 - 5.506; p=0.008, OR=0.504, 95% CI=0.303 - 0.83; p=0.01, OR=0.42, 95% CI=0.214 - 0.823, respectively). Judicial and medical history were also significant factors (p=0.004, OR=3.049, 95% CI=1.388 - 6.695; p=0.028, OR=0.446, 95% CI=0.214 - 0.93, respectively).

A history of multiple psychiatric hospitalizations was prevalent (p=0.005, OR=0.0597, 95% CI=0.0213 - 0.1678). Appointment regularity showed no significant association with adherence (p=0.045, OR=1.0294, 95% CI=0.7024 - 1.5112). The mean age of disease onset was 25.5 years, with first-generation antipsychotics and combination therapy being significant factors. Sedation, anticholinergic side effects, and libido disorders were common treatment side effects (p=0.036, OR=0.1174, 95% CI=0.0608 - 0.2272; p=0.0081, OR=1.2, 95% CI=0.8037 - 1.7919; p=0.013, OR=0.0588, 95% CI=0.0288 - 0.1195, respectively). Poor insight was prevalent among non-adherent patients (p=0.046, OR=0.1378, 95% CI=0.0786 - 0.2415). The average PANSS score during hospitalization was 69.7, indicating a broad spectrum of symptom severity (Table 1).

**Table 1:** associated factors to non-adherence among individuals with schizophrenia, 2018 (n=320)

Variable	Frequency (n=320)	Percentage (%)	p-value	ORs (95% CI)
**Age**			**0.003**	
<26	78	24.38		0.9615 (0.5357-1.7264)
26-31	75	23.44		1.0641 (0.6033-1.8775)
31-38	83	25.94		1.0769 (0.6112-1.8993)
≥38	84	26.25		1.0769 (0.6112-1.8993)
**Educational level**			**0.015**	
None	102	33		1.1811 (0.1904-0.4922)
Primary school	106	32		1.0392 (0.4407-1.7086)
Middle school	70	22		0.6863 (0.5319-3.0917)
Baccalaureate	31	10		0.3039 (0.1735-0.7864)
College	11	3		0.2222 (0.0734-0.3774)
**Residence**			**0.031**	
Urban	252	79		0.2063 (0.3216-0.5214)
Rural	52	16		0.635 (0.1288 -0.8742)
Homeless	16	5		0.2818 (0.064- 1.2374)
**Personal history**				
Toxic	220	68.75	0.0001	3.259 (1.929-5.506)
Tobacco	153	64.29	0.008	0.504 (0.303 -0.837)
Alcohol	69	28.99	0.01	0.42 (0.214- 0.823)
Cannabis	122	51.26	0.0001	0.231 (0.261- 0.421)
Judicial	67	20.94	0.004	3.049 (1.388 -6.695)
Medical	34	10.625	0.028	0.446 (0.214- 0.930)
**Previous hospitalizations**			**0.005**	
Never	29	9		0.167(-0.926-0.592)
One time	117	37		3.507(-0.526-1.540)
Several times	174	54		3.495 (0.121-6.870)
**Living environment**			**0.001**	
Alone	97	40.76		0.1443 (0.0728 -0.2879)
Parents	97	40.76		0.1444 (0.3487-0.5622)
Spouse	17	7.14		0.1924 (0.0477 -0.7785)
Family member	25	10.50		0.2191 (0.0621 -0.7735)
Institution	2	0.84		0.0218 (0.0003- 0.1610)
**Regularity of follow-up**			**0.045**	
Regular	138	56.87		2,0588 (1.3335- 3.2129)
Non-regular	182	43.13		0.0424 (0.0333-0.0536)
**Diagnosis criteria**				
Delusions	239	74.69	0.006	0.473 (0.274–0.816)
Hallucinations	237	74.06	0.007	2.48 (1.266-4.858)
**Age of onset**			**0.002**	
15-18	33	10.31		-1.099 (-1.655-0.543)
19-30	227	70.94		-1.193 (-1.479-0.907)
>30	60	18.75		-0.267 (-0.710-0.176)
**Number of drugs**			**0.0001**	
Monotherapy	72	22.5		-0.090 (-0.368-0.188)
Combination therapy	149	46.56		1.025(0.729-1.321)
More	99	30.49		3.093(2.440-3.746)
**Type of drugs**				
Typical	190	59.37	0.019	1.14 (1.08-2.46)
Atypical	128	40	0.004	0.51 (0.32-0.81)
Long-acting injectable (LAI)	70	21.87	0.010	2.72(1.26-5.89)
**Frequency of drug/day**			**0.0001**	
Once	57	17.81		0.108 (-0.314-0.530)
Twice	109	34.06		-0.465 (-0.784- -0.146)
Thrice	154	48.12		-2.538 (-3.143- -1.933)
**Side effects**				
Sedation	238	75	0.036	0.179 (-2.114- -1.326)
Weight gain	121	37.81	0.0081	12.234 (1.672-3.338)
Libido disorders	180	56.25	0.013	0.38(-3.877- -2.666)
Anticholinergic symptoms	224	70	0.015	0.184 (-2.156- -1.230)
**Insight**			**0.046**	
Poor	196	82.35		0.172(-1.841- -0.305)
Good	28	11.76		4.121(1.778-8.464)
Very good	14	4.20		4.605 (0.787-8.423)
**Positive and negative assesment scale**				
Postive	-	-	0.01	5.429 (0.845- 9.013)
Negative	-	-	0.01	11.265 (1.639 - 20.891)

## Discussion

Three hundred and twenty patients suffering from schizophrenia participated in the current study, and according to the results, 74% or 238 are non-adherent to their treatment, the finding is consistent with the results of literature reviews [10,11]. The current estimate is larger than the result from studies conducted in France (30%) [12] Nigeria [13] and India [14] successively achieving a non-adherence rate of 40% and 47%. This might be because of the difference in study design, study setting, study year, socio-demographic data, and type of screening tool used. In addition, non-adherence was reported to be linked to gender in this study, while Riecher-Rössler *et al*. [15]. Sajatovic *et al*. [16] found that women had better adherence than men, while the 2017 study of three Latin American countries concluded to the fact that poor adherence was more related to gender [17].

Gender differences in treatment efficacy and tolerance may stem from variations in physiological response, though conflicting findings persist in biomedical research [18-21]. The comparison of the age distribution in our study is statistically significant; younger patients tend to be poorly adherent compared to older patients. Sridhar *et al*. [14] found that non-adherent patients were younger than adherent. The results of a cross-sectional study that analyzed information obtained through interviews and surveys of patients diagnosed with schizophrenia attending public mental health centers in three Latin American cities: La Paz (Bolivia), Arica (Chile), and Tacna (Peru) [17] showed the same results as the current study.

In Nepal, young age was a risk factor for non-adherence to treatment, and this finding is in addition to several other studies [22,23]. This may be explained by the use of toxic substances by young people, or by their underestimation of the extent and severity of the disease. On the other hand, advanced age is a factor of poor adherence according to other studies [24,25]. Decreased cognitive abilities or impaired memory may be the cause of difficulties in older people. As for marital status, it does not seem to be a risk factor leading to non-adherence, which is moreover the case in our study. The studies by Yang *et al*. [26] and Wang *et al*. [27] did not find a significant correlation between adherence and the marital status of patients included in their studies. According to other studies, living alone appears to be a risk factor for medication non-adherence [28,29].

Substance misuse is a predictor of non-adherence to antipsychotic treatment in people diagnosed with schizophrenia, particularly cannabis, alcohol, and tobacco use, and international studies significantly link this factor to non-adherence [30,31]. Our study significantly links lack of adherence with medical, substance use history. A study in Thailand found that less than 40% of patients adhered to their antipsychotic treatment, resulting in an increased risk of hospitalization and annual healthcare costs [32]. The result of this study is similar to our work, with a highly significant (P=0.005), non-compliance increase in the rate of hospitalization of patients with schizophrenia. Our findings are consistent with several other studies that have found an association between poor adherence and a risk of hospitalization [33]. There is a strong relationship between age of onset and adherence in our study (p=0.0001); the early age of onset of symptoms is a risk factor [4]. Besides the severity of symptoms according to Perkins is proportional to the degree of non-adherence and this can be explained by the mistrust expressed by the patient towards any therapeutic action [34].

In a prospective study with a two-year follow-up period of patients with psychotic disorders at initial hospitalization, a higher baseline intensity of delusions and suspicion was associated with non-adherence at subsequent follow-up [35,36]. Schizophrenia is the psychiatric entity in which disorder awareness (or insight) is most frequently impaired. In our study, 48.78% of adherent patients have good insight, while 82.35% of non-adherent patients present poor insight. According to the study by Novick *et al*. [37] lack of insight has an impact on medication adherence as well as on the quality of life of patients with schizophrenia. Symptom severity in our study was assessed using the PANSS scale, with higher scores in the non-adherent group compared to the adherent group, so there is a significant correlation between symptom severity in patients with schizophrenia and treatment adherence (0.02). Several studies have also correlated increases in positive [38,39] and negative PANSS scores [40] with non-adherence to medication. The severity of positive symptoms such as delusions, hallucinations, and aggressive behavior may reduce adherence due to paranoia or non-cooperation, while the severity of negative symptoms may impair adherence due to a loss of willingness or motivation to adhere to treatment.

The study found that non-adherent patients were more likely to be on triple therapy, potentially due to increased complexity leading to forgetfulness and side effects, highlighting the challenge of simplifying prescriptions for patient adherence. The complexity of the prescribed treatment remains a serious problem [41]. Moreover, 50% of non-adherent patients took their antipsychotic treatment three times a day. According to a Japanese-American study published in 2013, which significantly links non-adherence to the number of doses taken, reducing and managing the number of drugs and the number of doses taken could improve patient adherence [41]. Speaking of antipsychotics, it seems to be that their use is responsible for various side effects, some embarrassing, and others life-threatening. These effects are greater in patients on first-generation antipsychotic drugs. Our study showed a close relationship between the experienced side effects and poor adherence, the main side effect generated by antipsychotic drugs is sedation with a significance level of P=0.036, followed by weight gain P=0.0081, libido disorders P=0.013, anticholinergic side effects with P=0.015. Several other studies link side effects to non-adherence [42,43].

The present study has some limitations. It was cross-sectional; therefore, the temporal relationship between risk factors and non-adherence cannot be determined. Although we've been assisted by psychiatry residents for data collection within the same hospital, it might have some information and recall biases. The patients´ report method used to measure treatment non-adherence might underestimate its magnitude compared to plasma concentrations.

## Conclusion

Schizophrenia is a chronic, disabling, and serious disease. The identification of the predictive factors that intervene and influence the compliance of treatment is a very important and unavailable for adequate management and a favorable evolution of this disease. The results of our study show that adherence to treatment was lower in young patients with more severe schizophrenic symptoms, with a lack of insight, the use of typical antipsychotics with a high number of intakes, and the side effects of treatment. The high number of hospitalizations is strongly correlated with a lack of medication adherence. According to our study, the factors found were the most involved in non-adherence, which should encourage healthcare teams to consider them to optimize medication adherence in patients suffering from schizophrenia.

### What is known about this topic

- *There have been many studies that have assessed the relapse rates of schizophrenia in regard to medication non-adherence, but few have been done in developing countries such as Morocco;*
- *This non-adherence may result in a more severe course of illness leaving patients with a higher level of disability;*
- *With the variation in the type of illness, education, and attitudes about treatments and outcomes, the perceptions of illness and its treatments can differ*.

### What this study adds

- *Non-adherence prevalence among individuals with schizophrenia at the University Psychiatric Center of Casablanca;*
- *Factors associated with non-adherence in this population;*
- *Use of scales to assess disease´s insight and severity*.
